# Factors associated with age-specific maternal health-seeking behaviours among women: A Multiple Indicator Cluster Survey-based study in 10 African countries

**DOI:** 10.7189/jogh.12.04095

**Published:** 2022-11-08

**Authors:** Qiwei He, Marhaba Abdureyim, Ziwei He, Xuemei Ma, Miaojia Huang, Tiange Zhang, Xinran Qi, Jiayi Hee, Kun Tang

**Affiliations:** 1 Vanke School of Public Health, Tsinghua University, Beijing, P.R. China; 2 Institute for Healthy China, Tsinghua University, Beijing, P.R. China; 3 School of Medicine, Tsinghua University, Beijing, P.R. China; 4 Institute for Hospital Management, Tsinghua University, Shenzhen, P.R. China; 5 Institute of Psychiatry, Psychology and Neuroscience, King’s College London, London, United Kingdom; 6 UWA Business School, The University of Western Australia, Perth, Australia; 7 School of Nursing and Rehabilitation, Shandong University, Jinan, P.R. China; 8 Department of Epidemiology, Johns Hopkins Bloomberg School of Public Health, Baltimore, United States

## Abstract

**Background:**

Maternal health-seeking behaviours (MHSB) are crucial for maintaining maternal health and reducing the maternal mortality ratio (MMR). However, little is known about age-specific MHSB in African countries. This study aims to examine the association between composite indicators of maternal characteristics, household conditions, and socioeconomic factors with MHSB among women from different childbearing age groups in 10 African countries.

**Methods:**

Based on the responses of 77 303 women and 68 391 households in 10 African countries to a nationally-representative round of the Multiple Indicator Cluster Survey (MICS6), we used age at childbearing to categorize women into groups according to their recent MHSB. In both pooled and age-specific analysis, multivariable logistic regression was applied to identify the predictors associated with MHSB. These factors were ranked with four sets of regression models.

**Results:**

This cross-sectional study found a prevalence of 27.69% (95% confidence interval (CI) = 26.93%-28.46%), 45.14% (95% CI = 44.29%-46.00%), and 28.60% (95% CI = 27.82%-29.40%) for four or more antenatal care visits (ANC4), intrapartum care (IPC), and postnatal care (PNC) service utilization, respectively. In the full sample, high household wealth ranked as the strongest determinant for all three MHSB, followed by mass media exposure for ANC4 utilization (odds ratio (OR) = 1.45; 95% CI = 1.20-1.76, *P* < 0.001), and higher education levels (secondary school education) for IPC and PNC utilization (IPC: OR = 1.49; 95% CI = 1.23-1.79, *P* < 0.001, PNC: OR = 1.39; 95% CI = 1.20-1.62, *P* < 0.001). However, higher maternal parity (three births and above) was associated with lower utilization of ANC4 (OR = 0.86; 95% CI = 0.76-0.96, *P* < 0.007), and residence in rural areas was associated with a lower IPC and PNC utilization (IPC: OR = 0.65; 95% CI = 0.54-0.79, *P* < 0.001, PNC: OR = 0.70; 95% CI = 0.57-0.85, *P* < 0.001).

**Conclusions:**

Our study provided further information on the direct and indirect factors associated with the utilization of maternal health services by women of different childbearing ages in 10 African countries. Additionally, the heterogeneous results among different childbearing age groups suggest that age-specific programmes and national policies are crucial for improving MHSB, and thus reducing MMR in Africa.

The United Nations Sustainable Development Goals (SDGs) recognize that ensuring individual health and promoting well-being is important for all people, regardless of age. Maternal health, a key part of SDG 3, is listed as its first target [1]. Recently, substantial progress has been made in reducing maternal deaths worldwide, with a 38% decline in the global maternal mortality ratio (MMR) from 2000 to 2017 [2]. However, maternal deaths remain alarmingly high globally; nearly 810 women die every day from preventable causes related to pregnancy or childbirth [3], though this number differs greatly between regions. According to the World Health Organization (WHO), 94% of all maternal deaths occur in low-resource settings such as those in African countries, which reported approximately 69% (202 700) of the estimated global maternal deaths (295 000) in 2017 [4]; thus, Africa was categorized as having an extremely high MMR (defined as more than 1000 maternal deaths per 100 000 live births).

The high MMR in Africa is affected by various factors, such as health expenditure, the availability and quality of maternal health services, and maternal health-seeking behaviours (MHSB) [5,6]. MHSB is defined as the utilization of maternal health services by pregnant individuals to ensure the health of themselves and their unborn children during pregnancy, childbirth, and the postpartum period [2,7]. MHSB includes antenatal care (ANC), intrapartum care (IPC), and postnatal care (PNC). There is broad agreement that improving MHSB is one of the key strategies for reducing the high MMR in African countries [8]. ANC facilitates the identification of risk factors and diagnosis of complications early in pregnancy, effectively reducing the MMR [9]. In Egypt, the MMR per 100 000 live births was reduced from 174 in 1992 to 84 in 2000 and later reduced to 55 in 2008 [10]. This reduction in the MMR was largely achieved through the utilization of ANC. Additionally, the WHO consistently updates the guidelines for postnatal care for mothers and new-borns, especially in low-resource settings, complementing recommendations on delivery techniques, new-born health care interventions, and others [11].

However, MHSB is not solely dependent on one determinant but several, including maternal household and regional conditions, maternal education, and mass media exposure [12]. For instance, maternal educational levels are an important determinant of health service utilization, as women who were able to read and write were almost five times more likely to seek ANC services than their counterparts[7,8]. Thus, identifying the determinants of MHSB in Africa is critical to improving the utilization of maternal health services and reducing the MMR worldwide.

Besides the mothers’ socioeconomic status, grouping them according to maternal age into groups below 19, 20-34, and above 35 years of age to conduct stratified research on maternal and child health can also be an important method [13,14]. Previous studies have shown that maternal age is a significant indicator affecting both antenatal and postnatal health-seeking behaviours, and is also associated with adverse pregnancy outcomes such as maternal and perinatal mortality [15], miscarriage, stillbirth [16], and systemic infections [17]. Adolescent mothers (aged 15-19) [18] and mothers with advanced maternal age (aged 35-49) [19] tend to demonstrate lower motivation to seek for continuum health services throughout pregnancy. During 2015-2020, Africa had the highest adolescent birth rate (ABR), estimated from the annual number of births per 1000 women aged 15-19 years measured in 201 countries and areas [20]. The ABR ranged from 111 births in Eastern Africa to 124 in Western Africa and 144 in Central Africa [21]. Although advanced maternal age (AMA) is also prevalent in African countries, where a high number of pregnancies is common as childbearing often continues until menopause [22], most AMA-specific studies are conducted in western high-income countries. Few studies have focused on MHSB among women of different childbearing age groups in Africa.

Given the importance of maternal health care utilization and the presence of region-specific knowledge gaps, this study aims to investigate the factors influencing MHSB across different ages in 10 African countries. Using data from the sixth round of the Multiple Indicator Cluster Survey (MICS6), we conducted pooled and age-specific analyses to provide practical evidence for interventions and to identify priorities for local policymakers, foreign aid donors, and international organizations to reduce the MMR in Africa.

## METHODS

### Data source and participants

**Figure 1:**
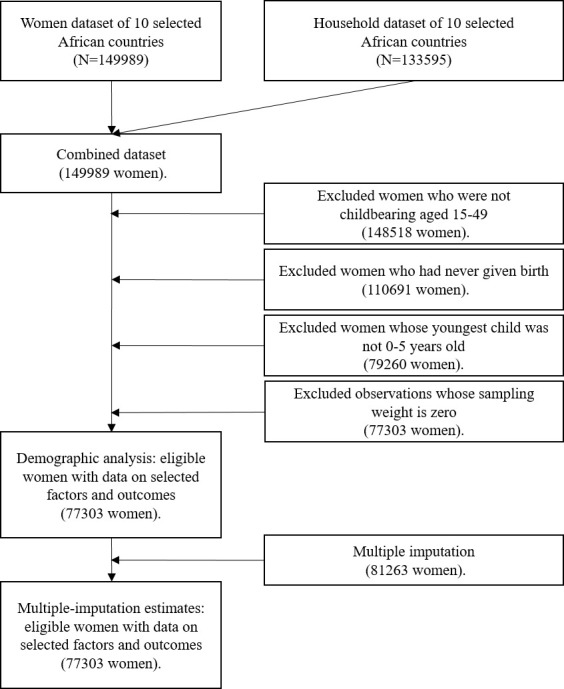
Flow diagram showing exclusions and final sample sizes of the study population, using the round six of Multiple Indicator Cluster Survey data.

We utilized data from the MICS6, a standardized household questionnaire that provides nationally representative and internationally comparable data on the living conditions of mothers and children from 2017 to 2019 [23]. We initially collected the complete data set of 15 African countries in MICS6 – Algeria, the Central African Republic, Chad, Costa Rica, the Democratic Republic of Congo, Gambia, Ghana, Guinea Bissau, Lesotho, Madagascar, Sao Tome and Principe, Sierra Leone, Togo, Tunisia, and Zimbabwe. However, we only utilized data from 10 African countries that had the complete data for all the variables of interest – the Central African Republic, Chad, the Democratic Republic of Congo, Guinea Bissau, Gambia, Ghana, Madagascar, Sierra Leone, Togo, and Tunisia. Data on MHSB variables and other variables of interest were retrieved for a total of 149 989 women and 133 595 households in these 10 African countries. Women 1) who were aged 15 to 49 years old, 2) who had ever given birth, 3) whose youngest child was zero to five years old at the time of the survey, and 4) whose sampling weight was not equal to zero were included in the study. All the sample weights are calculated and provided in MICS data sets to account for nonresponse, oversampling, and subsampling. To obtain our final analysis sample, we dropped the observations with zero sampling weight, which was automatically dropped in multivariable logistic regression model. Finally, we included a total of 77 303 women and 68 391 households from 10 African countries in the final analysis (**Figure 1**).

### Exposure

Following previous studies, we used 11 measures to determine MHSB [12,24].Variables of interest included childbirth experience (childbearing age, maternal parity, and age of the youngest child), maternal education and knowledge levels (educational attainment, HIV-related knowledge, and exposure to mass media), and marital status. We also included household factors such as the head of household’s sex, the head of household’s educational attainment, the household wealth index, and place of residence. Detailed definitions of each factor are provided in **Table 1**.

**Table 1:** Definition of 11 factors of maternal characteristics and household conditions associated with maternal health-seeking behaviour

Factors	Definition	Reference group
**Childbearing age**	A classified variable describing the age at the women gives birth to her last child. Calculated by woman’s documented birthday and the year of their last birth, and classified in the following three categories: 1) 15-19, 2) 20-34, 3) 35-49.	15-19
**Maternal parity**	A binary variable describing the amount of woman’s birth, and classified in the following two categories: 1) 1-2, 2) 3 and above.	1-2
**Age of youngest child**	A classified variable describing the age of women’s youngest child from zero to five years old. Classified in the following six categories: 1) 0, 2) 1, 3) 2, 4) 3, 5) 4, 6) 5.	0
**Maternal Educational attainment**	A classified variable describing women’s educational level in the three following categories: 1) no schooling or pre-primary school, 2) primary school education, 3) secondary or higher education.	No schooling or pre-primary school
**HIV-related knowledge**	A binary variable to reflect women’s awareness of HIV-related knowledge, and classified in the two following categories: 1) yes, if the woman was aware of HIV-prevention methods (ie, having only one partner who has no other sex partners is uninfected and who always uses a condom); knowing that a healthy person can be HIV positive; and disagreeing with HIV-related misinformation, 2) no, otherwise.	No otherwise
**Exposure to mass media**	A binary variable describing if women have access to mass media, and classified in the two following categories: 1) yes, if a woman used any mass media (including television, newspapers or magazine, or radio) at least once a week, 2) no, otherwise.	No otherwise
**Marital status**	A binary variable describing women’s marital condition. Based on the survey launched by UNICEF, marital status classified in the two 2 following categories: 1) married, 2) unmarried but living with a partner or single.	Unmarried but living with a partner or single
**Head of household’s sex**	A binary variable describing the biological sex of women’s household head in the two following categories: 1) male, 2) female.	Male
**Head of household’s educational attainment**	A classified variable describing women’s educational level using the three following categories: 1) no schooling or pre-primary school, 2) primary school education, 3) secondary or higher education.	No schooling or pre-primary school
**Household wealth index**	A classified variable describing household wealth level by ascertained through a selected set of household assets (computer, television, telephone, and internet, and classified in the five following categories: 1) the poorest, 2) second, 3) middle, 4) fourth, 5) the richest.	The poorest
**Place of residence**	A binary variable describing the women’s place of resident in the two following categories: 1) urban, 2) rural.	Urban

HIV – human immunodeficiency virus, UNICEF – United Nations Children Fund

### Outcomes

An operational continuum of care is one of the main factors in preventing pregnancy-related complications and decreasing the MMR [25]. The utilization of ANC, IPC, and PNC are integral parts of MHSB, so we followed previous research in coding the outcome variables. The WHO currently recommends a minimum of eight scheduled ANC visits (ANC8) [26]. Formerly, the recommended frequency was a minimum of four scheduled ANC visits (ANC4). However, given the low ANC attendance in Africa, we utilized the previous standard (ANC4) as our outcome variable [5]. ANC4 was defined as “Yes” and coded as “1” if the women had attended antenatal care at least four times during the last pregnancy. Otherwise, ANC4 was defined as “No” and coded as “0”. In addition to ANC, having a skilled attendant at the birth and giving birth in a health institution are also important for the continuum of care, according to previous research by the WHO and other authoritative agencies [26,27]. Hence, the presence of a skilled attendant at birth and institutional delivery, which have been adopted as leading indicators of maternal health by several international agencies [28], were incorporated into IPC. IPC was defined as “Yes” and coded as “1” if the women gave birth in health institutions (hospitals, clinics or health centres, health posts, and other health care centres) in the last birth, or had a skilled health professional (doctor, nurse, or midwife) assist with the last delivery. Otherwise, IPC was defined as “No” and coded as “0”. PNC was comprised of the umbilical cord examination, assessment of the infant’s temperature, and breastfeeding recommendations within 48 hours after birth. PNC was defined as “Yes” and coded as “1” if the infant’s umbilical cord was examined, someone examined the infant’s temperature, or someone counselled the mother on breastfeeding, within 48 hours after the last birth. Otherwise, PNC was defined as “No” and coded as “0”. Detailed definitions of each outcome are provided in **Table 2**.

**Table 2:** Definition of three outcomes that determine maternal health-seeking behaviour

MHSB outcomes	Definition	Category
**ANC4**	A binary variable, ANC4, was generated to indicate women’s ANC utilization during their last pregnancy, which contains blood pressure measurement, and urine and blood sample test. For women who had attended antenatal care at least four times during the last pregnancy, ANC4 was defined as “Yes” and coded as “1”. Otherwise, ANC4 was defined as “No” and coded as “0”.	1) yes, 2) no
**IPC**	A binary variable. For women who 1) gave birth in health institutions (hospitals, clinics or health centres, health posts, and other health care centres) in the last birth, or 2) had a skilled health professional (doctor, nurse, or midwife) assisted with the last delivery, IPC was defined as “Yes” and coded as “1”. Otherwise, IPC was defined as “No” and coded as “0”.	1) yes, 2) no
**PNC**	A binary variable, was defined as “Yes” and coded as “1” if 1) the infant’s umbilical cord was examined, 2) someone examined the infant’s temperature, or 3) someone counselled the mother on breastfeeding within 48 h after the last birth. Otherwise, PNC was defined as “No” and coded as “0”.	(1) yes, 2) no

ANC4 – minimum of four scheduled antenatal care, IPC – intrapartum care, PNC – postnatal care

### Statistical analysis

We examined the association of each factor of interest with MHSB outcomes in different age groups by pooling data from 10 African countries. To ensure that these estimates were sufficiently representative in both pooled analysis and age-specific analysis, we applied sampling weights and performed clustering according to the guidelines provided by the MICS6 [29]. Consistent with the methods of a previous study, the sample was clustered at the primary sampling unit level to allow for the interdependence of error terms within clusters and households [30]. We also re-weighted observations in both analyses according to the population size of each country and included each country’s fixed effects to explain unobservable national-level factors. Moreover, we treated the age of a woman’s youngest child as a time-fixed effect to control for the average differences across years. In the pooled analysis, we utilized multivariable logistic regression modelling to investigate the factors affecting ANC, IPC, and PNC. The models were adjusted for maternal educational attainment, HIV-related knowledge, exposure to mass media, maternal parity, childbearing age, the youngest child’s age, and marital status, as well as the head of household’s educational attainment, the head of household’s sex, the household wealth index, place of residence, and country, as appropriate. We then stratified women by childbearing age (dividing them into the following three age groups: 15-19 years old, 20-34 years old, and 35-49 years old) and repeated the analyses. Based on the above four sets of regression models, we ranked the factors according to their coefficient sizes (odds ratios (ORs)).

We used Stata version 16 (StataCorp LLC, College Station TX, USA) for all data analyses. Multiple imputations were used for missing values to increase the validity of this research. The level of statistical significance was set at 5% (*P* < 0.05) for all statistical analyses.

## RESULTS

### Descriptive statistics

As shown in **Table 3**, the majority of women were between 20 and 34 years old. The proportions of women who were married and had more than three prior births, no formal education, no prior HIV-related knowledge, and no mass media exposure were 69.84%, 61.41%, 43.14%, 84.35%, and 59.52%, respectively. A total of 69.78% of the women lived in rural areas. 27.04% women were in households belonging to the lowest wealth quintile, whereas 13.35% were in households in the highest wealth quintile. Regarding household heads, 79.62% were men, and 40.00% of the heads of household had no formal education. Overall, IPC utilization among women was the highest (45.14%; 95% confidence interval (CI) = 44.29%-46.00%), followed by PNC utilization (28.60%; 95% CI = 27.82%-29.40%) and ANC4 utilization (27.69%; 95% CI = 26.93%-28.46%). The prevalence of the use of all services (ie, ANC4, IPC, and PNC) was higher in women who had a lower parity (ie, one to two births), were more educated, lived in urban areas, were in highest wealth quintile households, had HIV-related knowledge, were exposed to mass media, had a head of household who was female, and had a head of household who had attained a relatively higher level of education.

**Table 3:** Distribution of maternal health-seeking behaviour by selected factors among women with childbearing age being 15 to 49 y old, using the sixth round of Multiple Indicator Cluster Surveys pooled across 10 African countries

		Prevalence, % (95% CI)		
**Factor**	**Women observed, n (%)**	**ANC4**	**IPC**	**PNC**
**Total sample for pooled analysis across 10 countries**	77 303	27.69% (26.93%-28.46%)	45.14% (44.29%-46.00%)	28.60% (27.82%-29.40%)
**Childbearing age**				
15-19	10 426 (13.49)	28.78% (26.65%-31.02%)	46.29% (43.94%-48.65%)	31.62% (29.42%-33.90%)
20-34	51 431 (66.53)	28.70% (27.77%-29.65%)	46.53% (45.49%-47.57%)	29.24% (28.29%-30.21%)
35-49	15 446 (19.98)	23.98% (22.38%-25.66%)	40.28% (38.40%-42.20%)	24.91% (23.20%-26.70%)
**Maternal parity number**				
1-2	29 832 (38.59)	32.32% (31.07%-33.60%)	48.36% (46.99%-49.73%)	32.56% (31.28%-33.87%)
3 and above	47 471 (61.41)	24.93% (23.99%-25.91%)	43.23% (42.14%-44.33%)	26.25% (25.27%-27.26%)
**Maternal education attainment**				
No schooling	33 348 (43.14)	20.48% (19.37%-21.65%)	34.30% (32.95%-35.68%)	22.60% (21.44%-23.81%)
Primary	22 568 (29.19)	24.47% (23.23%-25.74%)	43.48% (42.00%-44.97%)	26.27% (24.96%-27.62%)
Secondary	21 385 (27.66)	34.82% (33.45%-36.23%)	53.44% (51.97%-54.91%)	34.28% (32.88%-35.70%)
Missing	2 (0.00)	0% (0%-0%)	100.00% (100.00%-100.00%,)	23.50% (1.88%-83.08%)
**HIV-related knowledge**				
No	65 205 (84.35)	26.72% (25.92%-27.54%)	43.62% (42.72%-44.53%)	27.73% (26.90%-28.58%)
Yes	12 098 (15.65)	32.36% (30.23%-34.54%)	52.51% (50.21%-54.79%)	32.81% (30.65%-35.05%)
**Marital status**				
Unmarried	23 298 (30.14)	27.39% (26.04%-28.78%)	42.71% (41.17%-44.27%)	29.13% (27.72%-30.57%)
Married	53 989 (69.84)	27.82% (26.90%-28.75%)	46.21% (45.19%-47.23%)	28.37% (27.43%-29.33%)
Missing	16 (0.02)	21.87% (5.37%-58.01%)	34.49% (8.14%-75.77%)	19.32% (4.67%-53.94%)
**Mass media exposure**				
No	46 009 (59.52)	22.26% (21.37%-23.17%)	42.75% (41.69%-43.82%)	24.55% (23.62%-25.52%)
Yes	31 294 (40.48)	37.38% (36.01%-38.77%)	49.41% (47.98%-50.84%)	35.82% (34.45%-37.22%)
**Head of household’s sex**				
Male	61 545 (79.62)	27.50% (26.66%-28.36%)	44.87% (43.91%-45.83%)	28.44% (27.57%-29.32%)
Female	15 758 (20.38)	28.31% (26.60%-30.09%)	46.06% (44.19%-47.93%)	29.16% (27.40%-30.99%)
**Head of household’s education attainment**				
No schooling	30 921 (40.00)	23.61% (22.35%-24.92%)	34.34% (32.92%-35.78%)	26.13% (24.79%-27.52%)
Primary	20 415 (26.41)	24.07% (22.75%-25.44%)	40.80% (39.24%-42.39%)	25.58% (24.19%-27.03%)
Secondary	25 787 (33.36)	31.21% (30.03%-32.42%)	51.94% (50.63%-53.25%)	31.19% (29.98%-32.42%)
Missing	180 (0.23)	45.33% (27.37%-64.60%)	50.28% (32.55%-67.93%)	30.72% (19.30%-45.12%)
**Household wealth index**				
The poorest	20 904 (27.04)	22.52% (21.17%-23.92%)	38.90% (37.24%-40.58%)	22.65% (21.27%-24.09%)
Second	17 936 (23.20)	25.65% (24.12%-27.23%)	41.15% (39.47%-42.87%)	25.18% (23.65%-26.78%)
Middle	15 484 (20.03)	26.84% (25.23%-28.57%)	44.62% (42.71%-46.55%)	27.52% (25.82%-29.29%)
Fourth	12 657 (16.37)	30.12% (28.31%-31.99%)	51.08% (49.06%-53.10%)	32.31% (30.41%-34.27%)
The richest	10 322 (13.35)	35.03% (32.87%-37.26%)	51.97% (49.65%-54.29%)	37.51% (35.29%-39.78%)
**Place of residence**				
Urban	23 364 (30.22)	33.67% (32.23%-35.15%)	53.48% (51.90%-55.05%)	36.17% (34.66%-37.71%)
Rural	53 939 (69.78)	24.35% (23.49%-25.23%)	40.50% (39.50%-41.50%)	24.38% (23.51%-25.28%)
**Youngest child age**				
0	11 012 (14.25)	35.07% (33.03%-37.15%)	64.42% (62.68%-66.12%)	39.47% (37.41%-41.58%)
1	21 814 (28.22)	44.97% (43.39%-46.56%)	75.22% (74.15%-76.27%)	45.94% (44.35%-47.54%)
2	18 076 (23.38)	34.53% (32.86%-36.23%)	52.16% (50.45%-53.86%)	34.63% (32.92%-36.38%)
3	12 134 (15.70)	3.91% (3.57%-4.29%)	3.93% (3.59%-4.29%)	3.77% (3.43%-4.14%)
4	8245 (10.67)	3.14% (2.86%-3.45%)	3.36% (3.06%-3.69%)	3.16% (2.88%-3.47%)
5	6022 (7.79)	1.46% (1.24%-1.72%)	1.55% (1.33%-1.81%)	1.48% (1.26%-1.74%)

CI – confidence interval, ANC4 – minimum of four scheduled antenatal care, IPC – intrapartum care, PNC – postnatal care, HIV – human immunodeficiency virus

### Pooled analysis

**Figure 2:**
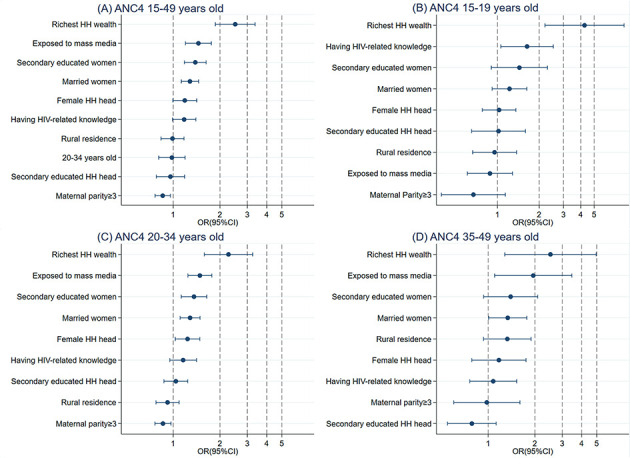
Relative ranking of factors associated with antenatal care from the full sample and age-specific model. Panel A: Utilization of ANC4 in women who bore a child at 15-49 years of age. Panel B: Utilization of ANC4 in women who bore a child at 15-19 years of age. Panel C: Utilization of ANC4 in women who bore a child at 20-34 years of age. Panel D: Utilization of ANC4 in women who bore a child at 35-49 years of age. Each row contains odds ratios (ORs) and 95% confidence intervals (CIs). HH – household, ANC4 – minimum of four scheduled antenatal care.

**Figure 3:**
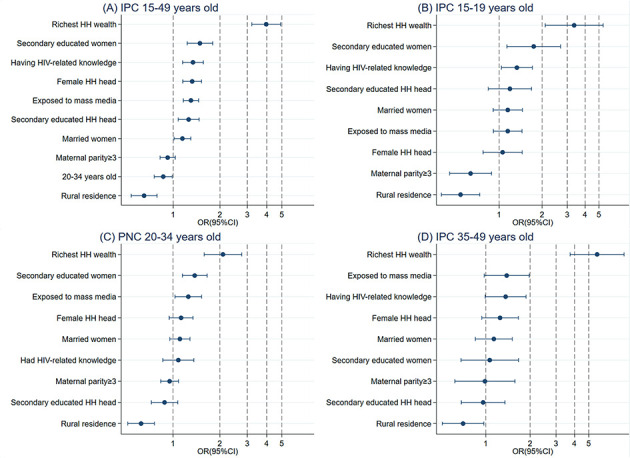
Relative ranking of factors associated with intrapartum care from the full sample and age-specific model. Panel A: Utilization of IPC in women who bore a child at 15-49 years of age. Panel B: Utilization of IPC in women who bore a child at 15-19 years of age. Panel C: Utilization of IPC in women who bore a child at 20-34 years of age. Panel D: Utilization of IPC in women who bore a child at 35-49 years of age. Each row contains odds ratios (ORs) and 95% confidence intervals (CIs). HH – household, IPC – intrapartum care.

**Figure 4:**
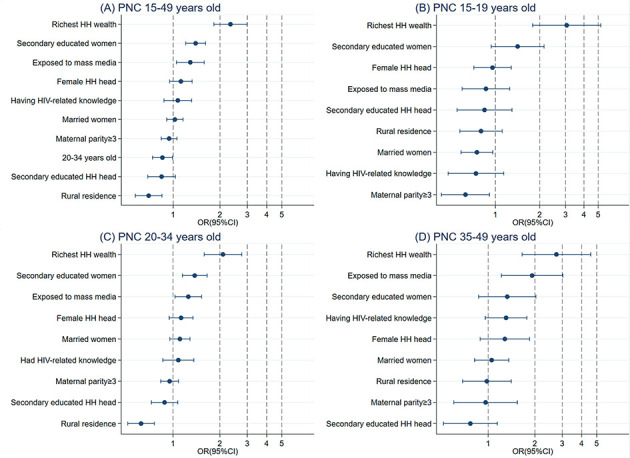
Relative ranking of factors associated with postnatal care from the full sample and age-specific model. Panel A: Utilization of PNC in women who bore a child at 15-49 years of age. Panel B: Utilization of PNC in women who bore a child at 15-19 years of age. Panel C: Utilization of PNC in women who bore a child at 20-34 years of age. Panel D: Utilization of PNC in women who bore a child at 35-49 years of age. Each row contains odds ratios (ORs) and 95% confidence intervals (CIs). HH – household, PNC – postnatal care.

The full sample and age-specific regression results are presented in **Table 4**, and all factors associated with ANC4, IPC and PNC are ranked in **Figure 2** (Panels A-D), **Figure 3** (Panels A-D), and **Figure 4** (Panels A-D), respectively.

**Table 4:** Magnitudes of factors associated with maternal health-seeking behaviour in the full sample, 15-19-, 20-34-, and 35-49-y-old childbearing age groups

Factor	ANC4, OR (95% CI)				IPC, OR (95% CI)				PNC, OR (95% CI)			
	**Full sample (15-49)**	**Group 1 (15-19)**	**Group 2 (20-34)**	**Group 3 (35-49)**	**Full sample (15-49)**	**Group 1 (15-19)**	**Group 2 (20-34)**	**Group 3 (35-49)**	**Full sample (15-49)**	**Group 1 (15-19)**	**Group 2 (20-34)**	**Group 3 (35-49)**
**Childbearing age (reference group: 15-19)**												
20-34	0.98 (0.81-1.19)†				0.86 (0.76-0.99)†				0.85 (0.74-0.99)†			
35-49	0.92 (0.72-1.18)				0.89 (0.74-1.07)				0.86 (0.71-1.04)			
**Maternal parity number (reference group: 1-2)**												
3 and above	0.86 (0.76-0.96)‡	0.67 (0.39-1.14)	0.86 (0.76-0.97)†	0.98 (0.60-1.60)	0.92 (0.82-1.03)	0.63 (0.45-0.89)‡	0.95 (0.84-1.08)	0.99 (0.62-1.58)	0.94 (0.84-1.06)	0.63 (0.43-0.91)†	0.95 (0.83-1.08)	0.96 (0.60-1.54)
**Maternal education attainment (reference group: no schooling)**												
Primary	1.10 (0.95-1.27)	1.01 (0.67-1.50)	1.14 (0.95-1.37)	1.07 (0.79-1.45)	1.24 (1.08-1.42)‡	1.60 (1.19-2.16)‡	1.22 (1.02-1.46)†	1.13 (0.89-1.45)	1.25 1.08-1.44)‡	1.34 (0.96-1.87)*	1.19 (1.01-1.41)†	1.35 (0.97-1.88)*
Secondary	1.39 (1.18-1.63)‡	1.44 (0.90-2.30)	1.36 (1.13-1.64)‡	1.4 (0.93-2.08)	1.49 (1.23-1.79)‡	1.75 (1.13-2.69)†	1.59 (1.29-1.95)‡	1.06 (0.68-1.67)	1.39 (1.20-1.62)‡	1.42 (0.94-2.14)	1.38 (1.15-1.66)‡	1.33 (0.87-2.03)
**HIV-related knowledge (reference group: no)**												
Yes	1.18 (0.99-1.40)*	1.64 (1.06-2.53)†	1.16 (0.95-1.41)	1.08 (0.76-1.53)	1.34 (1.15-1.56)‡	1.33 (1.04-1.71)†	1.34 (1.12-1.60)‡	1.36 (0.99-1.88)*	1.07 (0.87-1.31)	0.74 (0.48-1.14)	1.08 (0.86-1.36)	1.3 (0.96-1.78)*
**Marital status (reference group: unmarried)**												
Married	1.28 (1.13-1.45)‡	1.22 (0.91-1.63)	1.28 (1.11-1.49)‡	1.34 (1.01-1.77)†	1.15 (1.01-1.30)†	1.15 (0.91-1.45)	1.15 (1.01-1.30)†	1.13 (0.85-1.51)	1.03 (0.91-1.16)‡	0.75 (0.58-0.96)†	1.11 (0.95-1.29)	1.06 (0.82-1.36)
**Mass media exposure (reference group: no)**												
Yes	1.45 (1.20-1.76)‡	0.88 (0.61-1.28)	1.48 (1.24-1.77)‡	1.95 (1.10-3.46)†	1.30 (1.16-1.45)‡	1.15 (0.91-1.45)	1.32 (1.13-1.54)‡	1.39 (0.98-1.97)*	1.29 (1.05-1.58)†	0.86 (0.59-1.25)	1.25 (1.03-1.53)†	1.92 (1.22-3.02)‡
**Head of household’s sex (reference group: male)**												
Female	1.19 (1.00-1.42)*	1.03 (0.78-1.36)	1.24 (1.03-1.48)†	1.17 (0.78-1.75)*	1.32 (1.15-1.52)‡	1.06(0.77-1.45)	1.41 (1.21-1.65)‡	1.25 (0.94-1.66)	1.12 (0.95-1.32)	0.96 (0.71-1.28)	1.13 (0.94-1.34)	1.28 (0.89-1.85)
**Head of household’s education attainment (reference group: no schooling)**												
Primary	0.94 (0.79-1.12)	1.28 (0.92-1.80)	0.98 (0.82-1.17)	0.71 (0.51-1.00)*	1.19 (1.02-1.40)‡	0.96 (0.71-1.30)	1.27 (1.06-1.52)†	1.12 (0.82-1.52)	0.89 (0.74-1.07)	0.88 (0.66-1.16)	0.90 (0.73-1.10)	0.91 (0.61-1.35)
Secondary	0.96 (0.78-1.18)	1.02 (0.65-1.59)	1.04 (0.87-1.24)	0.79 (0.55-1.13)	1.26 (1.08-1.47)‡	1.19 (0.84-1.69)	1.37 (1.14-1.64)‡	0.96 (0.68-1.35)	0.84 (0.69-1.03)	0.84 0.55-1.30)	0.88 (0.73-1.07)	0.77 (0.52-1.14)
**Household wealth index (reference group: the poorest)**												
Second	1.33 (1.15-1.55)‡	1.67 (1.24-2.25)‡	1.27 (1.06-1.51)‡	1.36 (0.93-2.00)	1.30 (1.12-1.50)‡	1.28 (0.92-1.80)	1.27 (1.09-1.47)‡	1.43 (1.07-1.92)†	1.25 (1.08-1.44)‡	1.21 (0.92-1.58)	1.24 (1.06-1.44)‡	1.33 (0.93-1.91)
Middle	1.47 (1.25-1.73)‡	1.68 (1.13-2.49)†	1.4 (1.14-1.72)‡	1.57 (1.13-2.19)‡	1.75 (1.47-2.09)‡	1.8 (1.28-2.55)‡	1.64 (1.33-2.01)‡	2.18 (1.62-2.92)‡	1.44 (1.19-1.73)‡	1.94 (1.33-2.83)‡	1.35 (1.12-1.62)‡	1.44 (1.08-1.92)†
Fourth	1.62 (1.32-2.00)‡	1.84 (1.14-2.96)†	1.59 (1.27-1.99)‡	1.52 (0.95-2.44)*	2.45 (2.04-2.94)‡	2.69 (1.86-3.88)‡	2.17 (1.77-2.68)‡	3.5 (2.53-4.83)‡	1.57 (1.22-1.99)‡	2.42 (1.71-3.43)‡	1.39 (1.09-1.77)‡	1.8 (1.13-2.84)†
The richest	2.51 (1.87-3.36)‡	4.26 (2.20-8.22)‡	2.27 (1.59-3.25)‡	2.52 (1.28-4.96)‡	3.97 (3.20-4.91)‡	3.35 (2.10-5.35)‡	3.72 (2.85-4.86)‡	5.7 (3.74-8.68)‡	2.34 (1.83-2.99)‡	3.05 (1.79-5.21)‡	2.09 (1.59-2.76)‡	2.75 (1.65-4.57)‡
**Place of residence (reference group: urban)**												
Rural	0.99 (0.83-1.17)	0.95 (0.66-1.37)	0.93 (0.78-1.09)	1.33 (0.93-1.89)	0.65 (0.54-0.79)‡	0.54 (0.39-0.73)‡	0.66 (0.53-0.81)‡	0.70 (0.51-0.97)†	0.70 (0.57-0.85)‡	0.80 (0.57-1.11)	0.62 (0.51-0.76)‡	0.98 (0.68-1.41)
**Year dummies**	Included	Included	Included	Included	Included	Included	Included	Included	Included	Included	Included	Included
**Country dummies**	Included	Included	Included	Included	Included	Included	Included	Included	Included	Included	Included	Included
**_cons**	0.44 (0.32-0.60)‡	0.46 (0.24-0.87)†	0.46 (0.34-0.63)‡	0.22 (0.10-0.47)‡	1.25 (0.95-1.64)	1.90 (1.14-3.15)†	1.04 (0.78-1.40)	0.78 (0.37-1.62)	0.84 (0.64-1.10)	0.63 (0.38-1.04)*	0.87 (0.65-1.16)	0.40 (0.20-0.83)†

HH – household, ANC4 – minimum of four scheduled antenatal care, IPC – intrapartum care, PNC – postnatal care, OR – odds ratio, CI – confidence interval

*P < 0.10.

†*P* < 0.05.

‡*P* < 0.01.

### ANC4 Utilization

In the full sample model, women in highest wealth quintile households showed the highest utilization of ANC4 (OR = 2.51; 95% CI = 1.87-3.36, *P* < 0.001), followed by those exposed to mass media (OR = 1.45; 95% CI = 1.20-1.76, *P* < 0.001), those with higher education level (secondary school education: OR = 1.39; 95% CI = 1.18-1.63, *P* < 0.001), and those who were married (OR = 1.28; 95% CI = 1.13-1.45, *P* < 0.001). However, a higher maternal parity was associated with a lower utilization of ANC4 (three births and above: OR = 0.86; 95% CI = 0.76-0.96, *P* < 0.007).

In the age-specific regression model, being in a household in the highest wealth quintile was the strongest predictor of ANC4 utilization in all three childbearing age groups – 15-19 (OR = 4.26; 95% CI = 2-20-8.22, *P* < 0.001), 20-34 (OR = 2.27; 95% CI = 1.59-3.25, *P* < 0.001), and 35-49 (OR = 2.52; 95% CI = 1.28-4.96, *P* < 0.001) years old. Having HIV-related knowledge was the second strongest predictor for ANC4 utilization in women whose age at childbearing was 15-19 years (OR = 1.64; 95% CI = 1.06-2.53, *P* = 0.027), while exposure to mass media was the second strongest predictor in women aged 20-34 years (OR = 1.48; 95% CI = 1.24-1.77, *P* < 0.001) and women aged 35-49 years (OR = 1.95; 95% CI = 1.10-3.46, *P* = 0.022). Moreover, marriage only affected the association with ANC4 utilization in women aged 20-34 years (OR = 1.28; 95% CI = 1.11-1.49, *P* = 0.001) and in those aged 35-49 years (OR = 1.34; 95% CI = 1.01-1.77, *P* = 0.045). Additionally, higher education levels (OR = 1.36; 95% CI = 1.13-1.64, *P* = 0.001), having a female head of household (OR = 1.24; 95% CI = 1.03-1.48, *P* = 0.022), and higher maternal parity (three births and above: OR = 0.86; 95% CI = 0.76-0.97, *P* = 0.012) were significantly associated with ANC4 utilization in women whose age at childbearing was 20-34 years. However, these factors did not affect the utilization of ANC4 in the other two age groups.

### IPC utilization

In the full sample model, all factors were significantly associated with the utilization of IPC. Similar to ANC, women from households in the highest wealth quintile showed the highest utilization of IPC (OR = 3.97; 95% CI = 3.20-4.91, *P* < 0.001), followed by those with higher education levels (secondary school education: OR = 1.49; 95% CI = 1.23-1.79, *P* < 0.001), with HIV-related knowledge (OR = 1.34; 95% CI = 1.15-1.56, *P* < 0.001), with a female head of household (OR = 1.32; 95% CI = 1.15-1.52, *P* < 0.001), exposed to mass media (OR = 1.30; 95% CI = 1.16-1.45, *P* < 0.001), whose head of household attained a higher education level (OR = 1.26; 95% CI = 1.08-1.47, *P* = 0.004), and who were married (OR = 1.15; 95% CI = 1.01-1.30, *P* = 0.030). However, a higher childbearing age (20-34 years old: OR = 0.86; 95% CI = 0.76-0.99, *P* = 0.031) and residence in rural areas (OR = 0.65; 95% CI = 0.54-0.79, *P* < 0.001) were associated with a lower utilization of IPC.

In the age-specific regression model, the significant factors were the same for women whose age at childbearing was 20-34 years as for the full sample. Interestingly, in women aged 35-49 years, only two factors, having a household in the highest wealth quintile (OR = 5.70; 95% CI = 3.74-8.68, *P* < 0.001) and residing in rural areas (OR = 0.70; 95% CI = 0.51-0.97, *P* = 0.032), were significantly associated with the utilization of IPC. Moreover, in women whose age at childbearing was 12-19 years, being in a household in the highest wealth quintile (OR = 3.35; 95% CI = 2.10-5.35, *P* < 0.001), higher education levels (OR = 1.75; 95% CI = 1.13-2.64, *P* = 0.011), having HIV-related knowledge (OR = 1.33; 95% CI = 1.04-1.71, *P* < 0.001), higher maternal parity (three births and above: OR = 0.63; 95% CI = 0.45-0.89, *P* = 0.008) and residence in rural areas (OR = 0.54; 95% CI = 0.39-0.73, *P* < 0.001) were significantly associated with IPC utilization.

### PNC utilization

In the full sample model, women whose households were in the highest wealth quintile (OR = 2.34; 95% CI = 1.83-2.99, *P* < 0.001), who had higher education levels (secondary school education: OR = 1.39; 95% CI = 1.20-1.62, *P* < 0.001), who were exposed to mass media (OR = 1.28; 95% CI = 1.05-1.58, *P* = 0.016), who had a higher childbearing age (20-34 years old: OR = 0.85; 95% CI = 0.74-0.99, *P* = 0.035) and who lived in rural areas (OR = 0.70; 95% CI = 0.57-0.85, *P* < 0.001) were significantly associated with the utilization of PNC.

In the age-specific regression model, the significant factors were the same for women whose age at childbearing was 20-34 years as for the full sample, except for childbearing age. In women whose age at childbearing was 35-49 years, only those in households in the highest wealth quintiles (OR = 2.75; 95% CI = 1.65-4.57, *P* < 0.001) and those exposed to mass media (OR = 1.92; 95% CI = 1.22-3.02, *P* = 0.005) significantly predicted PNC utilization. Women who had households in the highest wealth quintile (OR = 3.05; 95% CI = 1.79-5.21, *P* < 0.001), were exposed to mass media (OR = 1.92; 95% CI = 1.22-3.02, *P* = 0.005), and were married (OR = 0.75; 95% CI = 0.58-0.96, *P* < 0.022) were significantly associated with PNC utilization in the 15-19-year-old age group.

## DISCUSSION

Our findings demonstrated that household wealth, maternal education, place of residence, HIV-related knowledge, mass media exposure, marital status, and maternal parity were significant determinants of MHSB (ie, utilization of ANC, IPC, and PNC) among different age groups.

The strong association between household wealth and MHSB outcomes may indicate that maternal health services could be expensive in these 10 African countries. A prior study in Togo revealed that families of lower socioeconomic status (eg, lower household wealth, lower maternal education, and those in rural areas) could not afford institutional delivery, as individuals with low socioeconomic status who gave birth in a hospital could remain in debt for years [31]. A previous study [32] and data from the World Bank also suggest that more than a quarter of the population in eight countries in Sub-Saharan Africa (SSA) (Ghana, Kenya, Malawi, Niger, Senegal, Uganda, Tanzania, and Zambia) could be pushed into poverty by out-of-pocket payments for health services [33]. To remove financial barriers and improve equity of access, efforts should be geared towards ensuring that the costs of maternal health services are within the financial abilities of low-income families through waiving user fees and offering free facility-based delivery.

This study indicates that MHSB among women less than 35 years old was strongly influenced by their educational attainment. This is consistent with prior studies conducted in SSA, which showed that levels of education equal to secondary school and above were associated with higher utilization of maternal health care services [34,35]. Evidence has shown that female educational attainment can improve health literacy [36], defined as a person's capacity to obtain, process, and understand basic health information and the services needed to make appropriate health decisions [37]. Studies on health literacy and women's reproductive health have reported that health literacy plays an important role in women's information seeking and reproductive knowledge [38], which may subsequently impact their health behaviours and outcomes. For women of AMA, personal experiences and habits during their lifespan may play a more important role than educational attainment, thus weakening the influence of educational attainment on MHSB. However, further research is needed to confirm these hypotheses.

Furthermore, we found that HIV-related knowledge was a significant determinant for both ANC4 (in the 15-19-year age group) and IPC (in the full sample, 15-19-year age group, and 20-34-year age group) utilization, indicating that access to HIV-related knowledge may be provided in schools or other through interventions targeted towards young mothers, such as the Determined, Resilient, Empowered, AIDS-Free, Mentored, and Safe (DREAMS) programme [39] conducted by the United States Agency for International Development (USAID), which aims to reduce the rates of HIV among adolescent girls and young women in SSA. Additionally, possessing comprehensive and correct knowledge of HIV, such as the risk of mother-to-child transmission, may better motivate mothers to seek maternal health care services. Thus, focused efforts are needed to improve health education, especially education related to reproductive health and HIV provided to adolescent mothers, and to raise awareness of MHSB.

We also found that living in rural areas was negatively associated with IPC and PNC utilization. Previous studies in Ghana found similar results: geographical barriers were not only the most common reason for having no attendants at a birth [40] but also the cause of delayed delivery and prolonged labour, resulting in nearly 149 maternal deaths in health facilities in 2011 [41]. Access to maternal health care facilities continues to pose a challenge for residents in rural areas in Africa due to transportation-related barriers and poor primary health care (PHC). A study including data from 100 databases on 48 SSA countries and islands estimated that approximately 28% of women of childbearing age lived more than two hours away from the nearest public hospital and were geographically marginalized from health care facilities [42]. Additionally, women living in rural areas are often less educated and less independent than their urban counterparts. A previous report by the WHO Regional Office of Africa suggested that modern health care services leave the predominantly rural population woefully underserved, as the majority of services are clinic-based, physician-oriented, and urban-centred[43]. Since reducing the travel time to a health facility is crucial for motivating and encouraging women to utilize maternal health services, policymakers should rethink the design of the health system and prioritize improvements to PHC to ensure equal access to comprehensive maternal health care.

For women whose age at childbearing was greater than 19 years old, we found that mass media exposure played an essential role in MHSB. This may be attributed to the differences in lifestyles and mass media usage among women of different ages. Our results are in line with those of a previous study conducted in Ethiopia, which demonstrated that women exposed to mass media at least twice a week were more likely to utilize maternal health services than those with less exposure [44]. Moreover, research has shown that mass media exposure significantly influences maternal health awareness [45] and that advertising campaigns provided women (as well as their husbands) with maternal health knowledge [46]. Besides providing education, promoting maternal health awareness via mass media (such as online applications and social media platforms) may be an effective intervention strategy to improve MHSB among women aged 20 to 49 in these 10 African countries.

We found a statistically significant association between marital status and utilization of ANC4 and IPC among our full sample; specifically, married women were more likely to utilize these services. Previous studies in African countries have also shown similar results in the utilization of maternity services [47,48]. This disparity may be due to stigmatization [49] and marginalization [50] of premarital sex and pregnancies in single mothers; thus, women who conceive out of wedlock may be less motivated to attend health care facilities [51]. Our results also demonstrated that, among women whose age at childbearing was less than 19 years, marriage appeared to be a barrier to the utilization of PNC. Previous studies have shown that child marriage could decrease the utilization of postnatal services [52,53]. A possible explanation might be that, as child brides are usually solely dependent on their husbands and mothers-in-law, they have limited decision-making autonomy regarding their own health care, which negatively influences MHSB. These individuals may also face social isolation and information barriers [52]. Another possibility might be that these families may focus more on the health of the baby than on that of the mother and hence would allot their limited finances to the utilization of ANC4 and IPC rather than the utilization of PNC for mothers. Therefore, maternal health care programmes should target unmarried pregnant women by increasing community support for changing social and gender norms. For married adolescent girls, broader interventions that focus on improving their decision-making autonomy may help to increase their utilization of maternal services.

We also found that high maternal parity was one of the greatest barriers to MHSB for women whose age at childbearing was less than 35 years old. This may be because young multiparous mothers tend to rely on their experience in previous pregnancies and do not feel the need to obtain maternal health care services from facilities [54]. However, women of AMA are more likely to seek maternal health care services due to their increased risks. Considering the significant association between high maternal parity and poor pregnancy outcomes, such as obstetric complications and perinatal death [55], future programmes should devote more attention to young multiparous mothers.

### Limitations

This study has several limitations, the first being recall bias; as the MICS6 questionnaire relies on self-reported data, recall bias is expected regarding questions on women’s MHSB during their last pregnancy. Second, we utilized ANC4 as our antenatal outcome instead of ANC8 recommended by the current WHO guidelines, as we found an extremely low prevalence of ANC8 among women in these 10 African countries. Third, the use of cross-sectional data prevented us from drawing causal inferences. Furthermore, outcomes may have been affected by omitted factors that may have been associated with the utilization of maternal health services. For example, spousal communication, dependency, transport, access to roads, as well as accessibility to and capacity of health care facilities may have an impact on a woman’s decision to seek professional care for childbirth. However, because the availability and completeness of these variables in Africa were poor, they were not analysed in this study.

## CONCLUSIONS

Our research provided further information on the direct and indirect factors associated with the utilization of maternal health care services by childbearing women of different age groups in these 10 African countries. More attention should be given to women with low socioeconomic status, low HIV-related knowledge, less mass media exposure, and high maternal parity. Additionally, the heterogeneous results among different age groups suggest that age-specific programmes and national policies are crucial for improving MHSB and thereby reducing the MMR in Africa.
